# A case of adenocarcinoma arising in an ileal diverticulum resected by laparoscopic surgery

**DOI:** 10.1186/s40792-016-0257-z

**Published:** 2016-11-17

**Authors:** Ryo Ikeshima, Junichi Nishimura, Hidekazu Takahashi, Naotsugu Haraguchi, Taishi Hata, Tsunekazu Mizushima, Yuichiro Doki, Masaki Mori

**Affiliations:** Department of Gastroenterological Surgery, Graduate School of Medicine, Osaka University, Yamadaoka 2-2, Suita, Osaka 565-0871 Japan

**Keywords:** Small intestinal diverticulum, Adenocarcinoma, Laparoscopic surgery

## Abstract

Adenocarcinoma arising in an ileal diverticulum are very rare. A 66-year-old man was recognized to have high serum CEA level and periappendiceal polycystic tumor in CT findings. Colonoscopy showed no abnormality in the ileocecal mucosa. However, the patient was suspected of appendiceal adenocarcinoma by PET/CT, which revealed FDG uptake with SUVmax of 3.9 in the tumor, and underwent radial surgery by single-incision laparoscopic surgery. Intraoperative findings showed the mass in the mesenterium of the terminal ileum but not the abnormality of the appendix. The resected specimen revealed a cystic tumor of 45 mm on the back side of the intestinal tract. Pathological findings showed that the tumor lesion mainly consisted of mucinous adenocarcinoma was developing from the base of the ileal diverticula. The postoperative process was going well, and the patient left the hospital 14 days after the operation. The recurrence has not been evident 10 months after the operation.

## Background

Adenocarcinoma arising in an ileal diverticulum has been little reported, since not only small intestinal diverticulum (SID) but also adenocarcinoma in the small intestine are relatively rare [1]. In the current report, we describe a case of adenocarcinoma arising in an ileal diverticulum who underwent radical surgery by SILS.

## Case presentation

**Fig. 1:**
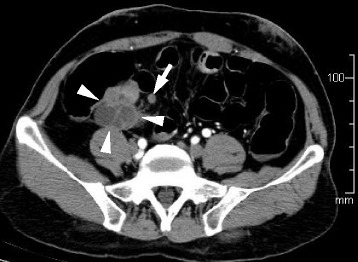
CT findings. Abdominal CT showed polycystic tumor in the ileocecal area (*arrowhead*) and a swollen lymph node (*arrow*)

**Fig. 2:**
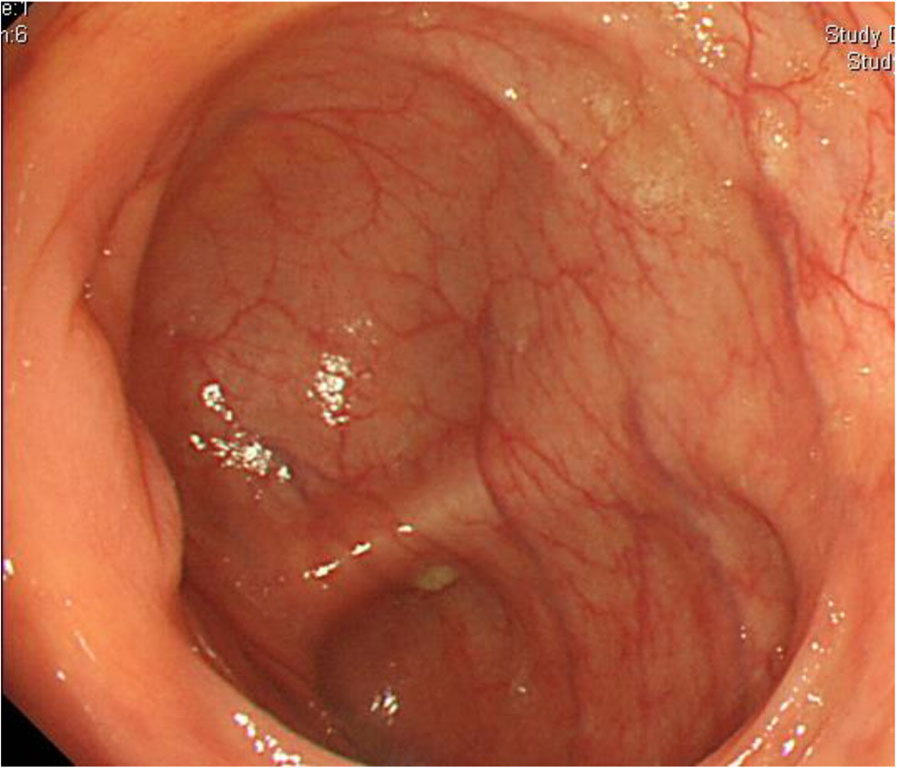
Endoscopic findings. The results of colonoscopy were normal in ileocecal mucosa. A biopsy of mucosa around the appendiceal orifice was done because of suspicion of appendiceal carcinoma in CT

**Fig. 3:**
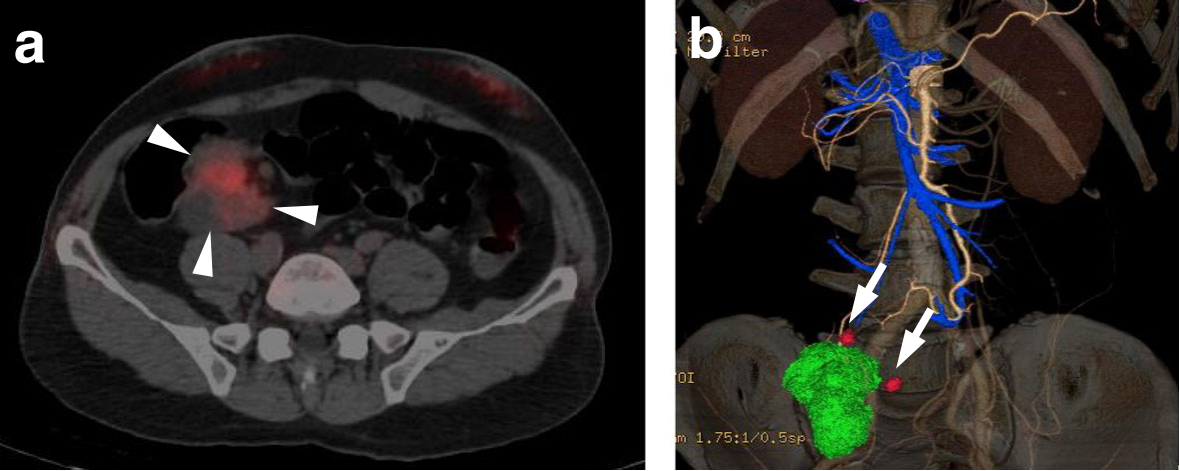
PET/CT colonography findings. **a** PET/CT showed polycystic tumor of 30 mm (*arrowhead*) with abnormal uptake on FDG-PET. **b** Lymph nodes neighboring the tumor were swelling (*arrow*)

A 66-year-old man had been under surveillance for diabetes, hypertension, and dyslipidemia. The patient was recognized to have high serum carcinoembryonic antigen (CEA) level, which let us suppose some digestive malignancies. On physical examination, no mass was palpable in his abdomen, and the patient had no symptoms like abdominal pain, vomiting, and nausea. Computed tomography (CT) revealed periappendiceal polycystic tumor of 40 mm and lymph node swelling of 9 mm neighboring the tumor (Fig. 1). Colonoscopy showed normal findings in the ileocecal mucosa (Fig. 2). A biopsy of the mucosa around the appendiceal orifice showed no abnormalities. PET/CT detected FDG uptake with SUVmax of 3.9 in the periappendiceal tumor (Fig. 3). Laboratory studies revealed an elevated serum concentration of not only CEA of 57 ng/ml but also carbohydrate antigen (CA) 19-9 of 4599 U/ml.

Thus, the patient was suspected to have appendiceal adenocarcinoma and underwent ileocecal resection. We selected SILS. Intraoperative findings showed the mass invading to the testicular artery and vein in the mesenterium of the terminal ileum and that the appendix stayed intact, but not showed the distinct metastasis, peritoneal dissemination, and ascites.

We achieved combined resection of the ileocecum and testicular artery and vein by SILS without tumor residual. The operation took a total of 195 min.

**Fig. 4:**
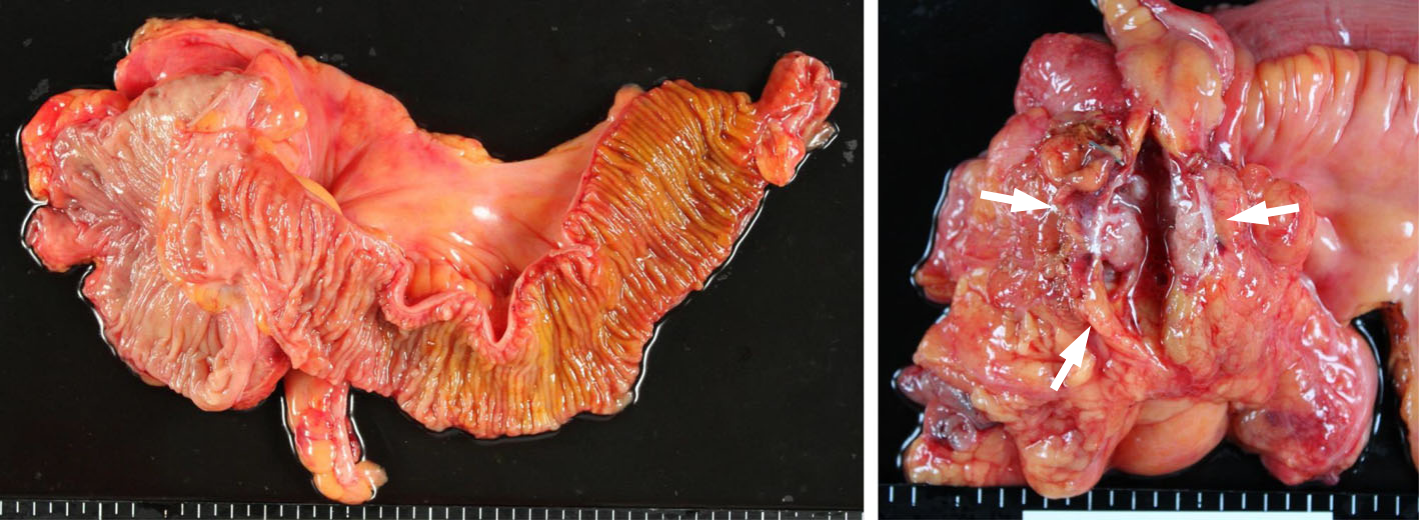
Gross findings. The resected specimen revealed no protruded lesion on mucosa of ileocecal but tumor on the back side of the intestinal tract. We made an incision to the tumor (*arrow*) and it revealed that the tumor was not solid but cystic

**Fig. 5:**
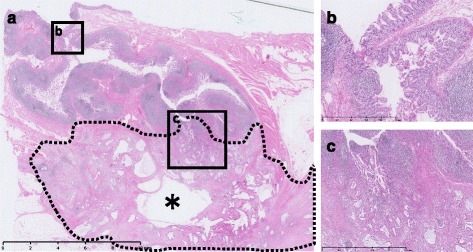
**a** Pathological findings showed diverticula under the ileocecal valve and cystic tumor including mucus (*asterisk*) was spreading from diverticula. **b** Ileal diverticulum protruding into the mesentery. **c** Mucinous, partly well-differentiated, adenocarcinoma was continuously from the base of the diverticulum

The resected specimen did not reveal any abnormal lesion on the ileocecal mucosa but a cystic tumor of 45 mm on the back side of the intestinal tract (Fig. 4). The pathological findings revealed a diverticulum under the ileocecal valve (Fig. 5a, b) and cystic tumor arising in its diverticulum, including mucus (Fig. 5c). The tumor lesion mainly consisted of mucinous adenocarcinoma and in part well-differentiated adenocarcinoma. Cancer cells had an invasion to subserosa but no lymph node metastasis. Further, cytological examination in mucus included in the tumor diagnosed as class V. And there was no invasion but adhesion to the testicular artery. Thus, we diagnosed mucinous adenocarcinoma arising in the ileal diverticulum (T3N0M0 stage IIA). The postoperative process was going well, and the patient left the hospital 14 days after the operation. The serum CEA and CA19-9 concentration got within normal limits. The tumor recurrence has not been evident 10 months after the operation.

SID excluding Meckel’s diverticula is only a few of all gastrointestinal diverticula [2], and the presence of them ranges from 0.1 to 1.5% in autopsy series [3–5]. SID consists of two subtypes. One is congenital true diverticulum in the contralateral mesentery and the other is acquired mostly pseudodiverticulum located in the mesenteric leaves [6]. About 10% of SID are required to perform surgery because of complications such as diverticulitis, bleeding, and perforation, but most of the SID are asymptomatic [7–9].

Small intestinal adenocarcinoma is also rare, constituting 1 to 2% of all primary gastrointestinal cancers [1]. Furthermore, adenocarcinoma of the ileum account for 15% of small intestinal adenocarcinoma, following the duodenum (55%) and the jejunum (30%) [10, 11]. Adenocarcinoma arising in a SID are extremely rare. A MEDLINE search showing only four cases was previously reported: two in a duodenal diverticulum [12, 13] and others in an ileal diverticulum [1, 14]. In spite of the similar incidence of non-Meckelian small intestinal diverticula and Meckel’s diverticula [15], adenocarcinoma arising in Meckel’s diverticula are reported much more than non-Meckelian diverticula [16–18]. In our case, adenocarcinoma was arising in the true diverticulum of the terminal ileum, so the patient was diagnosed with a case of non-Meckelian diverticulum.

Recently, advances of examination such as imaging and endoscopy are increasing small intestinal adenocarcinoma in a proper diagnostic rate. However, that is still difficult to diagnose because of absence of specific symptoms and signs and limited method of access to this organ. Among primary malignant neoplasms of the small intestine, carcinoid tumors are the most common histological types (41%), followed by adenocarcinomas (24%), lymphomas (22%), and sarcomas (11%) [19].

For accurate preoperative diagnosis of adenocarcinoma arising in the ileal diverticulum, strict examinations are important. Tsujii et al. [1] described a case of well- to moderately differentiated adenocarcinoma with neuroendocrine differentiation arising in an ileal diverticulum having high serum CEA level and normal concentrations of CA19-9 and that MRI, especially in coronal planes, appears to be a sensitive imaging modality for the diagnosis. And when it recurred in their case, the CEA level became elevated again. In our case, the patient was also occasionally found to have high CEA level by screening examination under surveillance for diabetes. Our patient’s tumor was mucinous, partly well-differentiated, adenocarcinoma. We did not undergo MRI and could not make a definitive diagnosis; however, as a result, we could have performed radical surgery by SILS for adenocarcinoma arising in an ileal diverticulum. CEA is capable of being an important factor to check the recurrence.

## Conclusions

The current authors encountered a case in which adenocarcinoma arising in an ileal diverticulum was performed in operation. Since it is very rare and mostly an asymptomatic disease, early detection and definite diagnosis are so difficult. There is a possibility that a high CEA level becomes a clue to diagnose; however, it needs accumulation of cases to reveal the possibility.

## Abbreviations

CA: Carbohydrate antigen
CEA: Carcinoembryonic antigen
CT: Computed tomography
MRI: Magnetic resonance imaging
SID: Small intestinal diverticula
SILS: Single-incision laparoscopic surgery
